# Targeted Next-Generation Sequencing for Clinical Diagnosis of 561 Mendelian Diseases

**DOI:** 10.1371/journal.pone.0133636

**Published:** 2015-08-14

**Authors:** Yanqiu Liu, Xiaoming Wei, Xiangdong Kong, Xueqin Guo, Yan Sun, Jianfen Man, Lique Du, Hui Zhu, Zelan Qu, Ping Tian, Bing Mao, Yun Yang

**Affiliations:** 1 Department of Genetics, Jiangxi Provincial Women and Children Hospital, Nanchang, 330006, China; 2 BGI-Wuhan, Wuhan, 430075, China; 3 BGI-Shenzhen, Shenzhen, 518083, China; 4 Prenatal Diagnosis Center, The First Affiliated Hospital of Zhengzhou University, Zhengzhou, 450052, China; 5 Department of Obstetrics and Gynecology, Wuhan Medical and Health Center for Women and Children, Wuhan, 430022, China; 6 Department of Neurology, Wuhan Medical and Health Center for Women and Children, Wuhan, 430022, China; Odense University hospital, DENMARK

## Abstract

**Background:**

Targeted next-generation sequencing (NGS) is a cost-effective approach for rapid and accurate detection of genetic mutations in patients with suspected genetic disorders, which can facilitate effective diagnosis.

**Methodology/Principal Findings:**

We designed a capture array to mainly capture all the coding sequence (CDS) of 2,181 genes associated with 561 Mendelian diseases and conducted NGS to detect mutations. The accuracy of NGS was 99.95%, which was obtained by comparing the genotypes of selected loci between our method and SNP Array in four samples from normal human adults. We also tested the stability of the method using a sample from normal human adults. The results showed that an average of 97.79% and 96.72% of single-nucleotide variants (SNVs) in the sample could be detected stably in a batch and different batches respectively. In addition, the method could detect various types of mutations. Some disease-causing mutations were detected in 69 clinical cases, including 62 SNVs, 14 insertions and deletions (Indels), 1 copy number variant (CNV), 1 microdeletion and 2 microduplications of chromosomes, of which 35 mutations were novel. Mutations were confirmed by Sanger sequencing or real-time polymerase chain reaction (PCR).

**Conclusions/Significance:**

Results of the evaluation showed that targeted NGS enabled to detect disease-causing mutations with high accuracy, stability, speed and throughput. Thus, the technology can be used for the clinical diagnosis of 561 Mendelian diseases.

## Introduction

At present, about 7000 Mendelian diseases have been recognized according to the Online Mendelian Inheritance in Man (OMIM) database. Although most of these diseases are individually rare, the total number of population affected by Mendelian diseases is vast. According to the data released by the World Health Organization (WHO), the global prevalence of all Mendelian diseases at birth is approximately 1:10000. The diagnosis is difficult for many Mendelian diseases and many are not onset at birth [1]. Except for some Mendelian diseases can be corrected by surgery, most are responsible for a heavy loss of life, disability or malformation. There was little relevant medicine to rare monogenic disorders [2]. It also lacks other effective treatment. Diagnostic testing can not only accurately determine whether a person carries the disease-causing gene or not, providing the effective basis for the diagnosis of the disease, but also estimate the risk of such a genetic disease in offspring, which plays an important role in genetic diagnosis and fertility guidance. So effective and exact diagnostic testing is very necessary.

Sanger sequencing is a common method of first generation of sequencing technology. Shortcomings such as narrow screening spectrum and poor stability of experiment limit the application in wide range screening of gene mutations. The primary advantage of NGS over Sanger method is the inexpensive production of large amounts of sequencing data [3]. With unprecedented high throughput, highly parallel and base-pair resolution data of NGS, whole-exome sequencing (WES) and whole-genome sequencing (WGS) are all applied in the diagnostic testing or carrier screening of Mendelian genetic disorders [4–7]. WGS and WES yield myriad of genetic variations which have an advantage in carrier screening. However, identifying the few disease-causing mutations among the vast variants present in human genomes remains a major challenge [8]. Targeted NGS with lower cost and high throughput can efficiently target disease-associated regions of the genome and detect variants with high sensitivity, which is widely used in the clinical screening of mutations and effective diagnostic testing of genetic diseases [9–12], including some cancers [13–15]. Therefore, we designed a capture array which could mainly capture the CDS of 2,181 genes associated with 561 Mendelian diseases and developed a pipeline including experiment, sequencing, bioinformatic analysis, mutation identification and confirmation.

## Materials and Methods

### Information of Samples

We totally collected 94 samples for this study. To evaluate the stability of our method, S1 was sequenced three times in a batch (S1-1, S1-2 and S1-3) and two times in different batches (S1-4 and S1-5). Four samples (S1-1, S2, S3 and S4) from normal human adults were selected to evaluate the accuracy of our method. In addition, we selected 84 patient samples and 6 samples (P44, P45, P77, P78, P81 and P82) of suspected carriers from clinical cases (S1 Table). Among them, 75 samples were diagnosed and 15 samples were undiagnosed (Table 1). Written informed consent was obtained from all of the adult participants or the parents of the minors enrolled in our study. This project and the protocols for the investigation involving human tissues were approved by the ethics committee of BGI-Shenzhen (BGI-IRB 14061).

**Table 1:** Summary of disease-causing mutations in 90 clinical cases.

ID	Sex, age^a^	Clinical diagnosis^b^	Candidate causative genes	Mutations^c^, status	Consistency^d^
P1	F, 4 years	AxD	*GFAP*	*GFAP* NM_001242376.1:c.236G>A (p.R79H)	Yes
P2	M, 23 days	AS	*COL4A4*, *COL4A5*, *et al*.	*COL4A5* NM_033380.2:c.1769A>C (p.K590T) (Hem), novel	Yes
P3	F, 6 years	AS	*COL4A4*, *COL4A5*, *et al*.	*COL4A5* NM_033380.2:c.2414G>T (p.G805V)	Yes
P4	F, 13 years	AS	*COL4A4*, *COL4A5*, *et al*.	*COL4A5* NM_033380.2:c.187G>T (p.G63*), novel	Yes
P5	M, 27 years	APS-1?	*AIRE*	negative result	No
P6	M, 37 years	ADPKD	*PKD1*, *PKD2*	*PKD1* NM_001009944.2:c.9914_9915delCT (p.A3305Pfs*10), novel	Yes
P7	F, 32 years	ADPKD	*PKD1*, *PKD2*	negative result	No
P8	F, 22 years	ADPKD	*PKD1*, *PKD2*	*PKD1* NM_001009944.2:c.7210-1G>A, novel	Yes
P9	M, 29 years	ADPKD	*PKD1*, *PKD2*	negative result	No
P10	M, 42 years	ADPKD	*PKD1*, *PKD2*	*PKD1* NM_001009944.2:c.1589G>A (p.C530Y)	Yes
P11	M, 32 years	ADPKD	*PKD1*, *PKD2*	negative result	No
P12	M, 74 years	ADPKD	*PKD1*, *PKD2*	negative result	No
P13	F, 39 years	ADPKD	*PKD1*, *PKD2*	*PKD1* NM_001009944.2:c.7210-2A>G	Yes
P14	F, 30 years	ADPKD	*PKD1*, *PKD2*	*PKD1* NM_001009944.2:c.8698C>T (p.Q2900*)	Yes
P15	M, -	ADPKD	*PKD1*, *PKD2*	*PKD1* NM_001009944.2:c.7204C>T (p.R2402*)	Yes
P16	M, 28 years	ADPKD	*PKD1*, *PKD2*	negative result	No
P17	M, 51years	ADPKD	*PKD1*, *PKD2*	negative result	No
P18	M, 30 years	ADPKD	*PKD1*, *PKD2*	negative result	No
P19	F, 28 years	ADPKD	*PKD1*, *PKD2*	negative result	No
P20	M, 33 years	ADPKD	*PKD1*, *PKD2*	*PKD1* NM_001009944.2:c.7566 C>A (p.C2522*), novel	Yes
P21	M, 67 years	ADPKD	*PKD1*, *PKD2*	*PKD1* NM_001009944.2:c.10618G>T (p.G3540*), novel	Yes
P22	M, 30years	ADPKD	*PKD1*, *PKD2*	negative result	No
P23	F, 30 years	ADPKD	*PKD1*, *PKD2*	*PKD1* NM_001009944.2:c.2085_2086insC (p.A696Rfs*18)	Yes
P24	F, 37 years	ADPKD	*PKD1*, *PKD2*	*PKD1* NM_001009944.2:c.9377C>T (p.T3126I)	Yes
P25	M, 32 years	ADPKD	*PKD1*, *PKD2*	*PKD1* NM_001009944.2:c.8350 C>T (p.Q2784*)	Yes
P26	M, 60 years	ADPKD	*PKD1*, *PKD2*	negative result	No
P27	M, 29 years	ADPKD	*PKD1*, *PKD2*	*PKD1* NM_001009944.2:c.9569G>T (p.G3190V), novel	Yes
P28	M, -	ADPKD	*PKD1*, *PKD2*	*PKD1* NM_001009944.2:c.11721_11722insCT (p.L3909Cfs*37), novel	Yes
P29	M, -	ADPKD	*PKD1*, *PKD2*	*PKD1* NM_001009944.2:c.11343C>A (p.Y3781*), novel	Yes
P30	F, 58 years	ADPKD	*PKD1*, *PKD2*	*PKD1* NM_001009944.2:c.6574_6580delACCGCCA (p.T2192Afs*18)	Yes
P31	M, -	ADPKD	*PKD1*, *PKD2*	*PKD1* NM_001009944.2:c.12608_12635delGGCTGGGGACAAGGTGTGAGCCTGAGCC (p.R4203Pfs*93)	Yes
P32	F, 27 years	ADPKD	*PKD1*, *PKD2*	*PKD1* NM_001009944.2:c.8578C>T (p.Q2860*), novel	Yes
P33	M, 31 years	ADPKD	*PKD1*, *PKD2*	*PKD1* NM_001009944.2:c.12003+2T>C	Yes
P34	F, 31 years	ADPKD	*PKD1*, *PKD2*	*PKD1* NM_001009944.2:c.8590G>T (p.E2864*), novel	Yes
P35	F, 28 years	ADPKD	*PKD1*, *PKD2*	*PKD1* NM_001009944.2:c.12373C>T (p.Q4125*)	Yes
P36	F, 26 years	ADPKD	*PKD1*, *PKD2*	*PKD1* NM_001009944.2:c.12373C>T (p.Q4125*)	Yes
P37	M, 26 years	ADPKD	*PKD1*, *PKD2*	*PKD1* NM_001009944.2:c.5014_5015delAG (p.R1672Gfs*98)	Yes
P38	M, 26 years	ADPKD	*PKD1*, *PKD2*	*PKD1* NM_001009944.2:c.8590G>T (p.E2864*), novel	Yes
P39	F, 27 years	ADPKD	*PKD1*, *PKD2*	*PKD1* NM_001009944.2:c.7915C>T (p.R2639*)	Yes
P40	M, 42 years	ADPKD	*PKD1*, *PKD2*	*PKD1* NM_001009944.2:c.4306C>T (p.R1436*)	Yes
P41	F,56 years	ADPKD	*PKD1*, *PKD2*	*PKD1* NM_001009944.2:c.7546C>T (p.R2516C)	Yes
P42	M, 32 years	ADPKD	*PKD1*, *PKD2*	*PKD1* NM_001009944.2:c.7546C>T (p.R2516C)	Yes
P43	-, 11 years	ARCI	*ALOX12B*, *ALOXE3*, *et al*.	*ALOXE3* NM_001165960.1:c.814C>T (p.R272*) (Hom), novel	Yes
P44	M, -	ARCI, carrier	*ALOX12B*, *ALOXE3*, *et al*.	*ALOXE3* NM_001165960.1:c.814C>T (p.R272*), novel	Yes
P45	F, -	ARCI, carrier	*ALOX12B*, *ALOXE3*, *et al*.	*ALOXE3* NM_001165960.1:c.814C>T (p.R272*), novel	Yes
P46	F, 27 years	BS	*SLC12A1*, *CLCNKB*, *et al*.	negative result	No
P47	F, 7 months	BS?	*BSND*, *KCNJ1*, *et al*.	*CFTR* NM_000492.3:c.1116+1G>A c.3062C>T (p.P1021L), novel	CF
P48	M, 6 months	BS?	*BSND*, *KCNJ1*, *et al*.	*CFTR* NM_000492.3:c.2909G>A (p.G970D) (Hom)	CF
P49	F, 33 years	CMT	*AARS*, *EGR2*, *et al*.	*AARS* NM_001605.2:c.2042G>T (p.G681V), novel	Yes
P50	F, 35 years	CMT	*AARS*, *EGR2*, *et al*.	*EGR2* NM_001136177.1:c.928ins>GCC (p.Y310delinsCH), novel	Yes
P51	F, 3 years	CMT	*AARS*, *EGR2*, *et al*.	negative result	No
P52	F, 24 years	CMT	*AARS*, *EGR2*, *et al*.	*PMP22* NM_153322.1:c.215C>T (p.S72L)	Yes
P53	M, 36 years	CMT	*AARS*, *EGR2*, *et al*.	*MFN2* NM_014874.3:c.1039-2A>G, novel; *GJB1* NM_000166.5:c.265C>G (p.L89V) (Hem), novel	Yes
P54	M, 28 years	CMT	*AARS*, *EGR2*, *et al*.	*GJB1* NM_000166.5:c.223C>T (p.R75W) (Hem)	Yes
P55	F, 12 years	CMT	*AARS*, *EGR2*, *et al*.	*GDAP1* NM_018972.2:c.767A>G (p.H256R)	Yes
P56	M, 10 years	CMT	*AARS*, *EGR2*, *et al*.	*PMP22* NM_153322.1:CDS1-4 dup	Yes
P57	M, 37 years	CMT	*AARS*, *EGR2*, *et al*.	*GJB1* NM_000166.5:c.269T>C (p.L90P) (Hem), novel	Yes
P58	F, 3.5 years	Congenital Afibrinogenemia	*FGA*, *FGB*, *FGG*	*FGA* NM_000508.3:c.1368delC (p.T457Rfs*27) (Hom), novel	Yes
P59	F, 5 years	CDA?	*CDAN1*, *SEC23B*, *KLF1*	*PKLR* NM_000298.5:c.1528C>T (p.R510*) c.661G>A (p.D221N)	PK Deficiency
P60	-, 3 years	CdLS	*NIPBL*, *SMC1A*, *et al*.	*NIPBL* NM_133433.3:c.2207_2211delCAAAG (p.Q738Rfs*2), novel	Yes
P61	M, 8 years	Dent Disease	*CLCN5*, *OCRL*	*OCRL* NM_000276.3:c.215_216delTT (p.L73Dfs*2) (Hem)	Yes
P62	M, 1 year	Dent Disease	*CLCN5*, *OCRL*	*CLCN5* NM_001127899.1:c.778_798delACTCTGGTTATCAAAACCATC (p.T260_I266del) (Hem), novel	Yes
P63	F, 18 months	FHL	*PRF1*, *STX11*, *et al*.	*PRF1* NM_005041.4:c.1620A>G (p.Q540Q)	Yes
P64	F, 34 years	GS	*SLC12A3*, *CLCNKB*	*SLC12A3* NM_000339.2:c.1202C>T (p.A401V) (Hom), novel	Yes
P65	-, -	GSD?	*AGL*, *ALDOA*, *et al*.	*GALT* NM_000155.3:c.1043A>G (p.D348G) (Hom), novel	Galactosemia
P66	M, -	Hereditary Muscular Disease?	*ACTA1*, *ATP1A3*, *et al*.	negative result	No
P67	M, 3 years	HSAN	*IKBKAP*, *NTRK1*, *et al*.	*NTRK1* NM_002529.3:c.963delG (p.L322Sfs*148), novel c.851-33T>A	Yes
P68	-, -	Hypochondroplasia?	*FGFR3*	*COL2A1* NM_001844.4:c.863G>C (p.G288A), novel; *LBR* NM_194442.2:c.1640A>G (p.N547S), novel c.1757G>A (p.R586H), novel	Yes
P69	M, 4 years	Hypophosphatasia	*ALPL*	*ALPL* NM_000478.4:c.98C>T(p.A33V)(Hom)	Yes
P70	F, 2 years	IP	*IKBKG*	negative result	No
P71	M, 75 days	IBD deficiency? EE? SCAD Deficiency?	*ACAD8*, *ETHE1*, *ACADS*	*ACADS* NM_000017.2:c.989G>A (p.R330H) c.1031A>G (p.E344G)	Yes
P72	M, 55 days	IBD deficiency? EE? SCAD Deficiency?	*ACAD8*, *ETHE1*, *ACADS*	*ACADS* NM_000017.2:c.164C>T(p.P55L) c.1031A>G(p.E344G)	Yes
P73	F, 41 years	LPG	*APOE*	*APOE* NM_000041.2:c.127C>T (p.R43C)	Yes
P74	M, -	MMA, carrier	*MUT*, *MMAA*, *et al*.	*MMACHC* NM_015506.2:c.656_658delAGA (p.K220del)	Yes
P75	F, -	MMA, carrier	*MUT*, *MMAA*, *et al*.	*MMACHC* NM_015506.2:c.609G>A (p.W203*)	Yes
P76	M, -	Microphthalmia	*SOX2*, *OTX2*, *et al*	*OTX2* NM_021728.3:c.538C>T (p.Q180*), novel	Yes
P77	F, 6 years	Mucopolysaccharidosis	*ARSB*, *GALNS*, *et al*.	*GNPTAB* NM_024312.4:c.3565C>T (p.R1189*) c.2590_2591insG (p.E864Gfs*4)	Mucolipidosis
P78	F, 28 years	Mucopolysaccharidosis, carrier?	*ARSB*, *GALNS*, *et al*.	negative result	No
P79	M, 31 years	Mucopolysaccharidosis, carrier?	*ARSB*, *GALNS*, *et al*.	negative result	No
P80	F, 33 years	OCA	*GPR143*, *TYR*, *et al*.	*TYR* NM_000372.4:c.455C>A (p.P152H), novel c.832C>T (p.R278*)	Yes
P81	F, 6 years	MCPH	*MCPH1*, *WDR62*, *et al*.	negative result	No
P82	M, 5 years	PCD?	*DNAI1*, *DNAH5*, *et al*.	negative result	No
P83	M, 8 years	SLSN	*CEP290*, *IQCB1*, *et al*.	*IQCB1* NM_001023570.2:c.1225C>T (p.Q409*), novel c.1090C>T (p.R364*)	Yes
P84	F, 9 years	SRNS?	*ACTN4*, *ALG1*, *et al*	negative result	No
P85	F, 12 years	SRNS?	*ACTN4*, *ALG1*, *et al*	negative result	No
P86	F, 11 months	WAS? X-SCID?	*WAS*, *IL2RG*	*RAG1* NM_000448.2:c.874T>C (p.S292P), novel c.1328G>A (p.R443K), novel	OS
P87	M, 14 years	XLA	*BTK*	*BTK* NM_000061.2:c.1684C>T (p.R562W) (Hem)	Yes
P88	-, -	Trisomy 10	-	47.XX,+10	Yes
P89	-, -	Trisomy 9	-	47.XX,+9	Yes
P90	-, 8 years	SMS	-	46,XN,del(17)(p11.2)	Yes

^a^Sex, age: ‘-’ shows that the sex or age of a sample is not available.

^b^Clinical diagnosis: The result of this column indicates the diagnosis of clinical cases in the form of abbreviations (S2 Table). The question mark indicates the case is undiagnosed and the result is noted with ‘carrier’ if the case is a suspected carrier.

^c^mutations: The results of this column is the summary of disease-causing mutations. Homozygous mutations are marked with (Hom) and hemizygous mutations are marked with (Hem), the other unmarked mutations are heterozygous. Mutation is noted with ‘novel’ if it is novel. ‘*’ indicates a termination codon.

^d^Consistency: Consistency is defined as ‘Yes’ if our result is consistent with the clinical diagnosis. Consistency is defined as ‘No’ if the result is negative. Consistency is noted with the name of the disease based on our analysis if we could not detect mutations in candidate causative genes but could detect mutations in other genes and our result is inconsistent with the clinical diagnosis.

### Chip Design, Experiment and Sequencing

A capture array (NimbleGen, Roche) was designed to mainly capture the CDS of 2,181 known pathogenic genes associated with 561 Mendelian diseases (S3 Table) based on GeneReviews (NCBI) and Genetics Home Reference.

Genomic DNA from peripheral blood or tissues of abortion was fragmented ranging from 200 bp to 250bp. The primers, adapters and indexes were then ligated to the DNA fragments to construct libraries. The DNA fragments were pooled and hybridized to the capture array (Roche NimbleGen, Inc.). After hybridization and enrichment, the DNA sample was sequenced on Illumina HiSeq2500 Analyzers to generate paired-end reads (90 bps). The detailed procedure is roughly the same as used in a previous paper [9].

### Bioinformatic Analysis and Mutation Identification

**Fig 1:**
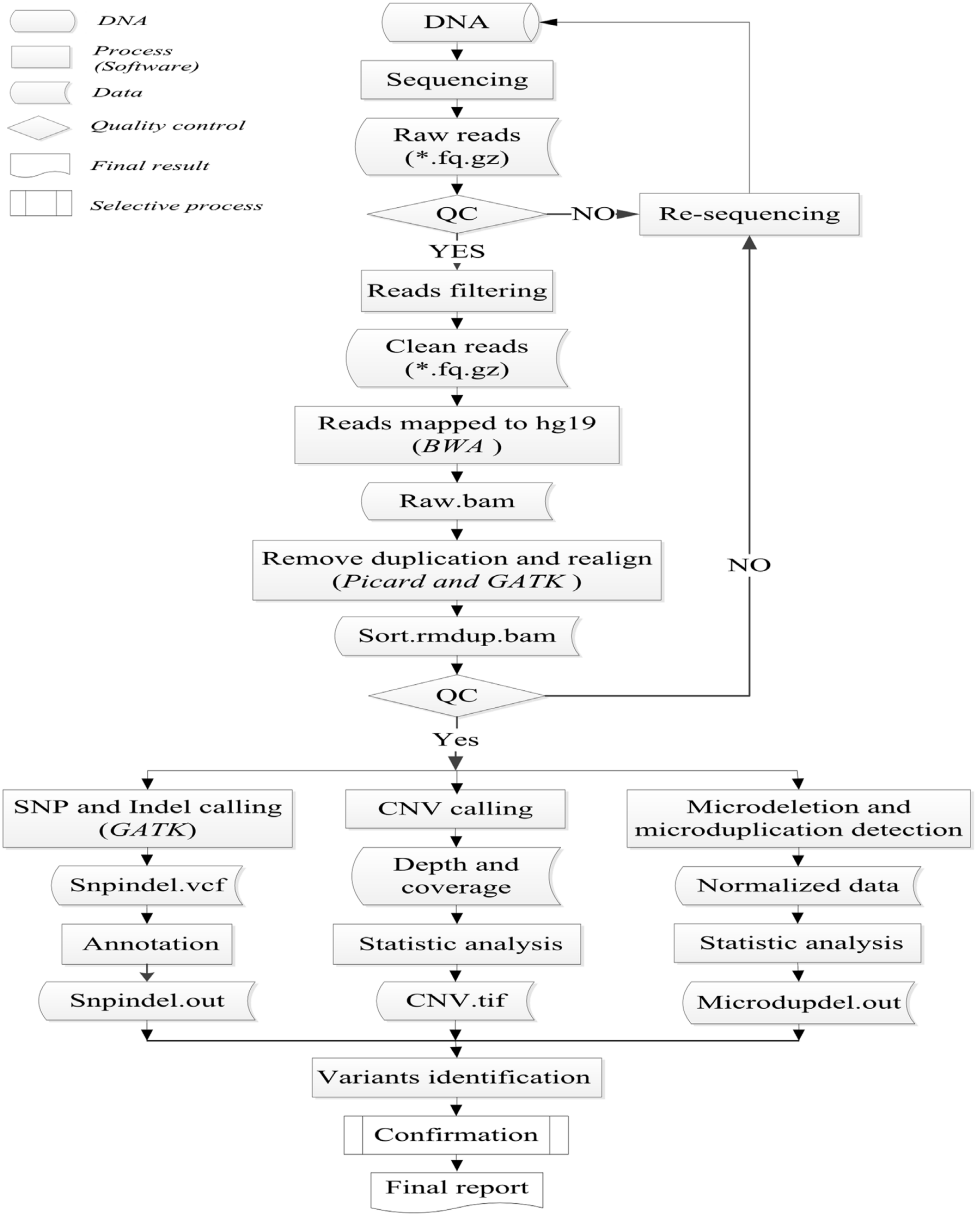
Flowchart for the process of bioinformatic analysis.

The pipeline of bioinformatic analysis included data filteration, data alignment, variants detection and results annotation (Fig 1). The pipeline started from the sequencing data (Raw reads) which generated from the HiSeq2500. First, after a preliminary quality assessment of sequencing data using the Illumina Pipeline (version 1.3.4), reads containing adapters and with low-quality values which defined as that the average quality value was less than ten were filtered out. Second, the clean reads were mapped to the human reference genome from the NCBI database (Build37) using Burrows Wheeler Aligner (BWA). Third, after removing duplications caused by PCR using Picard and realigning by Genome Analysis Toolkit (GATK), the bam results were used to do variants detection. Single-nucleotide polymorphisms (SNPs) and Indels were detected using GATK. CNVs were identified by a method based on comparison of average depth between patients and normal human samples in the same batch, which was introduced in detail in a previous paper [9]. Normalization analysis was a method to detect microdeletions and microduplications of chromosome using read count-based approach [16]. The microdeletions or microduplications of chromosomes related to our target regions were selected from the database of DECIPHER [17]. Finally, SNPs and Indels were annotated by databases such as dblocal (Mutation frequency database of 100 normal human samples) [9], dbSNP (http://www.ncbi.nlm.nih.gov/SNP/), HapMap (http://hapmap.ncbi.nlm.nih.gov/), dbNSFP (http://varianttools.sourceforge.net/Annotation/DbNSFP), HGMD (http://www.hgmd.org/) and the 1000 Genome (http://www.1000genomes.org/).

According to the annotation of the variants, we could obtain the frequency of occurrence and pathogenicity of the variants in different selected databases above. Based on the patient's clinical working diagnosis, candidate disease-causing genes could be found in our own database. According to the variants detected, we first focused on the variants in the candidate genes. If we could not find disease-causing mutations in the candidate genes, we would look for mutations in the other genes.

### Confirmation

To verify some SNVs and Indels detected by our method, Sanger sequencing (ABI 3730 DNA Analyzer; Applied Biosystems) was performed for three inconsistent genotypes in two samples (S1-1 and S4) and 5 pedigrees. To quantify duplications in the *PMP22* gene, real-time PCR (7500 Real-time PCR system, Applied Biosystems) was performed for patient P56.

## Results

### Analysis of Sequencing Data

The total size of target regions of the capture array is 6.19Mb. Except all the CDS of 2,181 genes associated with 561 Mendelian diseases, the target regions also included some disease-related introns and UTRs of the 2,181 genes and the immediately adjacent region of 10bps of each region. The DNA probes in the array can hybridize with all of the targeted regions and flanking sequences of 100 bps on each side of the target region.

To evaluate the technology and detect mutations in patients and suspected carriers, we obtained at least 6, 925, 841 reads (P72) mapped to target regions per sample. The reads length was 90bps, so we got at least 623.33M bases mapped to target region per sample. The average sequencing depth of each sample was above 77.43-fold (P72) and the highest sequencing depth was 277.43-fold (P32). At least 90.34% of target regions with more than 20-fold were successfully captured and the coverage of target region was at least 99.4% (S1 Table).

### Evaluation of Accuracy

**Fig 2:**
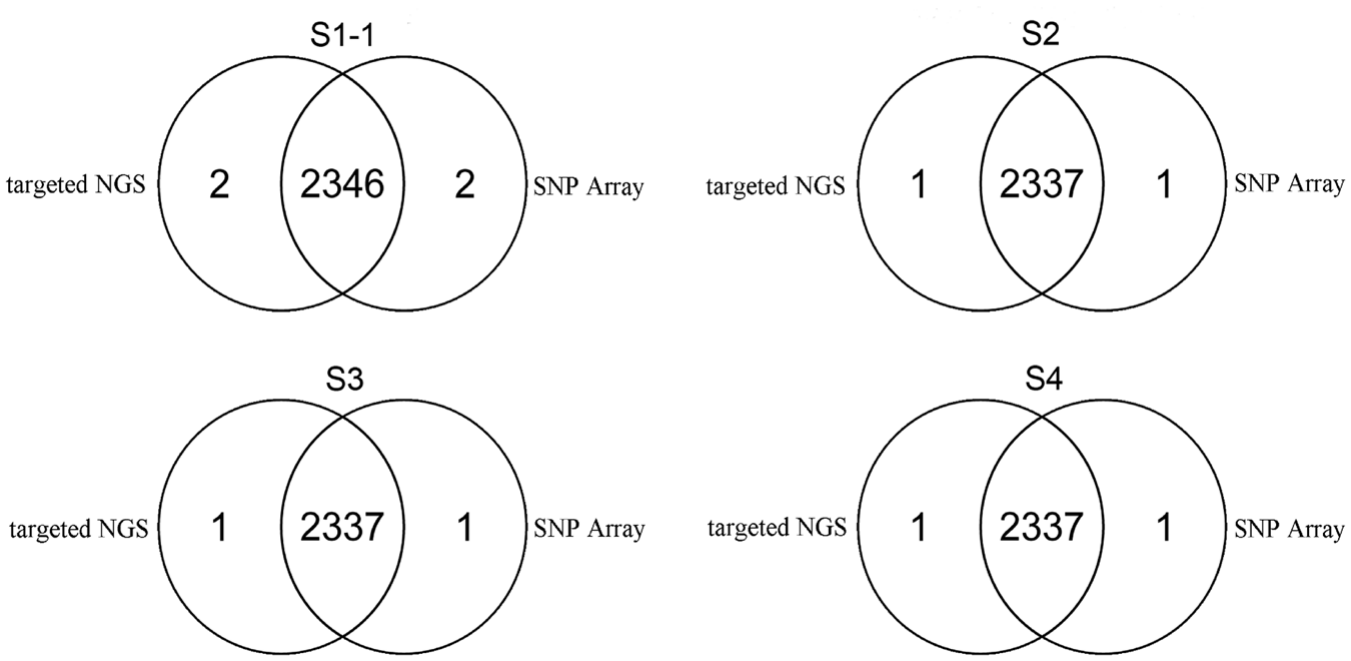
Evaluation of the accuracy of our method. Venn diagrams of the number of genotypes detected by targeted NGS and SNP Array in four samples (S1-1, S2, S3 and S4).

To assess the accuracy of the technology, we selected four samples (S-1, S2, S3 and S4) from normal human adults. The average depth of S1-1 was only 79.49-fold and the average depth of other three samples was about 200-fold (S1 Table). Genotypes were detected using targeted NGS and SNP Array (Illumina's Human Zhonghua-8 Bead Chips) respectively. In sample S1-1, 99.91% (2346/2348) genotypes of selected loci detected by targeted NGS were accordant to the genotypes detected by SNP Array. In each of the other three samples (S2, S3 and S4), 99.96% (2337/2338) genotypes of selected loci detected by targeted NGS were accordant to the genotypes detected by SNP Array (Fig 2). So the average consistency was 99.95% and the inconsistent genotypes (Table 2) were validated by Sanger sequencing. So, our method had a high accuracy even in the case of low average depth which was only about 80-fold.

**Table 2:** Inconsistent genotypes detected by our method and SNP Array.

Sample	Sex	Chr	Position	Rs number	Reference	Alleles of NGS	Alleles of SNP Array
S1-1	male	chr4	106158216	rs3796927	G	A/G	A
S1-1	male	chrX	8503641	rs809446	C	T	T/C
S2	female	chrY	14851554	rs2032599	-	-	T
S3	female	chrY	14851554	rs2032599	-	-	T
S4	male	chrX	8503641	rs809446	C	T	T/C

### Evaluation of Stability

**Fig 3:**
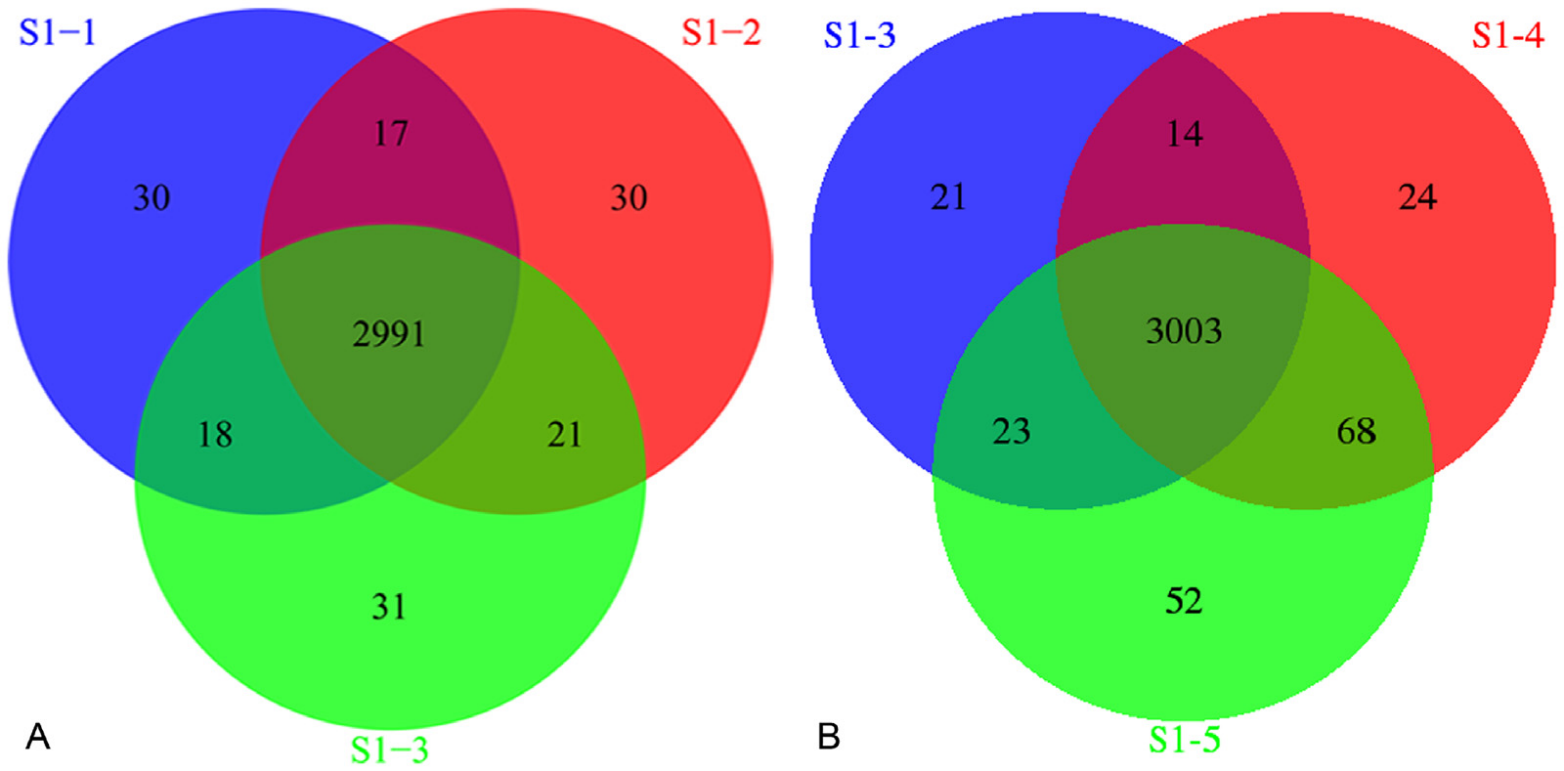
Evaluation of the stability of our method. (A) Venn diagram of S1 sequenced three times in a same batch. (B) Venn diagram of S1 sequenced three times in different batches.

To assess the stability of the technology, S1 were sequenced three times (S1-1, S1-2 and S1-3) in a batch and the average depth of each test was about 80-fold. S1 were sequenced two times (S1-4 and S1-5) in two different batches and the average depth of the two tests was 98.99-fold and 180.71-fold respectively (S1 Table). Due to most of target regions were CDS, SNVs in noncoding regions were incredible, so we only compared the SNVs in CDS. The total numbers of SNVs of three tests (S1-1, S1-2 and S1-3) in a batch were 3056, 3059 and 3061 respectively, the same SNVs of three tests were 2991, the proportion of same SNVs in each test were 97.87%, 97.78% and 97.71% respectively, with an average of 97.79%. The total number of SNVs of three tests (S1-3, S1-4 and S1-5) in three different batches were 3061, 3109 and 3146 respectively, the same SNVs of three tests were 3003, the proportion of same SNVs in each test were 98.11%, 96.59% and 95.45% respectively, with an average of 96.72% (Fig 3). So an average of 97.79% and 96.72% of SNVs can be stably detected in a same batch and different batches respectively, which indicated good stability of our method.

In addition, we also compared genotypes of S1-2, S1-3, S1-4 and S1-5 with that of SNP Array respectively. We found that there were only two inconsistent loci in each test which were the same as in S1–1 (Table 2). So, the accordance ratio of genotypes was all 99.91% (2346/2348) whether in a test from the same batch or not, showing high consistency, which also indicated good stability of our method.

### Variants Identification

Using targeted NGS, we obtained high-quality reads of 90 samples. After alignment and variants detection, the variants were obtained. Based on the annotation of the variants, some disease-causing mutations (62 SNVs, 14 Indels, 1 CNV, 1 microdeletion and 2 microduplications of chromosomes) were identified in 65 patients and 4 suspected carriers, 35 of which were novel. Table 1 summarized the total mutations of the 90 samples.

There were three couples of 6 suspected carriers (P44, P45, P74, P75, P78 and P79) from normal human adults. P44 and P45 are parents of patient P43 who was diagnosed with autosomal recessive Congenital Ichthyosis. An *ALOXE3* gene nonsense mutation (c.814C>T, p.R272*, #MIM 606545) was detected in the three subjects. The mutation was homozygous in the proband (P43) and heterozygous in his parents, which followed the segregation rules. The incidence of this mutation in human populations was extremely low based on annotated databases. According to database of OMIM, the *ALOXE3* gene mutations can cause autosomal recessive Nonbullous Congenital Ichthyosiform Erythroderma, so the mutation was causative for the proband. We detected a heterozygous *MMACHC* gene mutation (c.656_658delAGA, p.K220del, OMIM# 277400) in P74 and a heterozygous *MMACHC* gene nonsense mutation (c.609G>A, p.W203*, OMIM# 277400) in P75 who previously bore a child with Methylmalonic Acidemia. It was reported that the mutations c.609G>A could cause Methylmalonic Academia [18]. Although there was no literature reported about the mutation of c.656_658delAGA, the incidence in human populations was very low based on annotated databases. So the two mutations were suspected causative for the child. P78 and P79 had born a child who was dead with suspected Mucopolysaccharidosis VII, but we found no pathogenic mutations in them.

**Fig 4:**
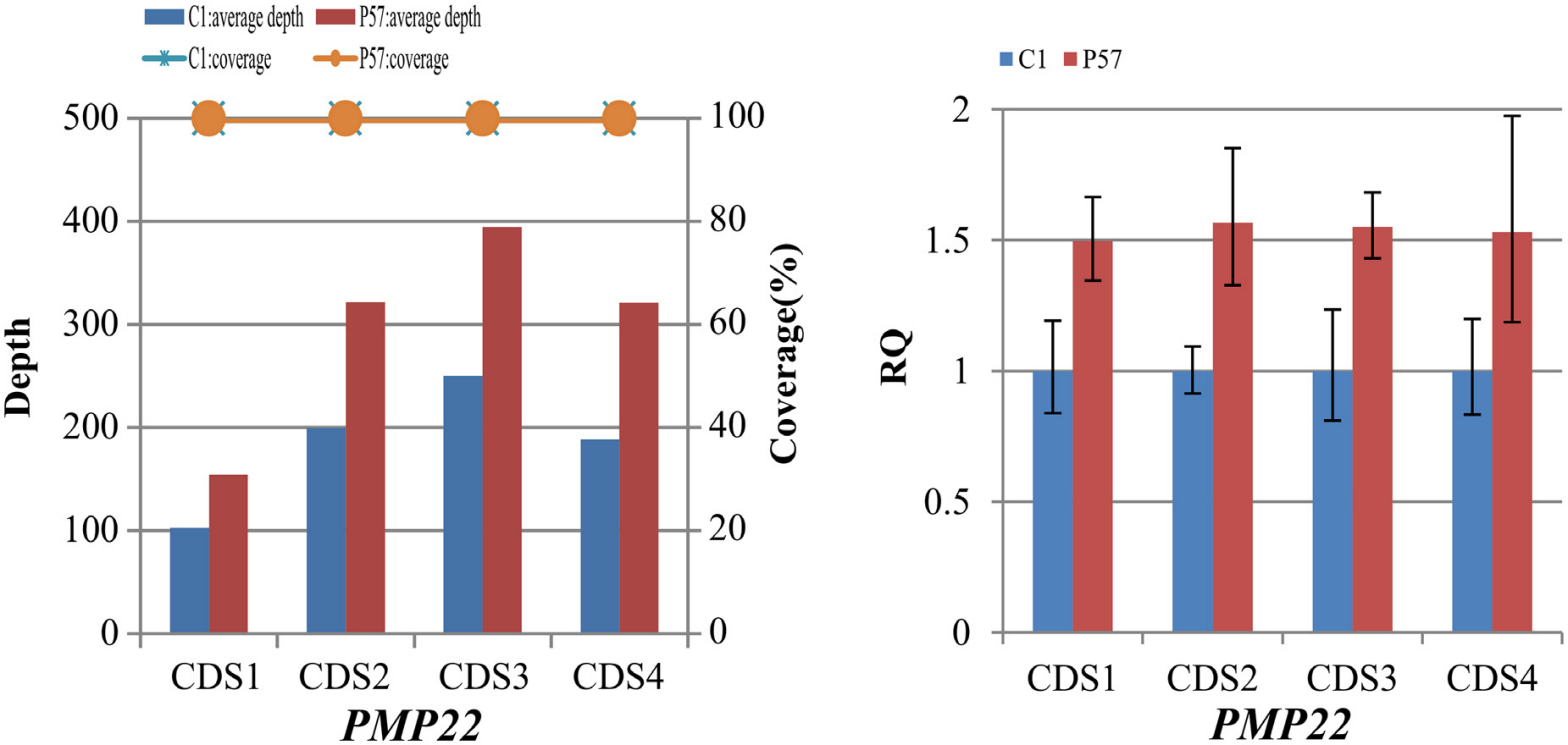
Identification and confirmation of heterozygous duplications involved in CMT in P57. (A) Diagram of the average sequencing depth and coverage for CDS1-4 in the *PMP22* gene. (B) Quantitative real-time PCR analysis. Relative amplification value calculated from the data of quantitative real-time PCR for detecting possible duplications in the *PMP22* gene of patient P57.

Based on the method of CNV detection above, we identified heterozygous duplications of CDS1-4 in the *PMP22* gene in patient P57 who were diagnosed with Charcot-Marie-Tooth (CMT) disease (Fig 4A). Duplication of *PMP22* gene could cause over expression of encoded protein and affect the normal function of cells which was the most common factor leading to CMT [19–21]. Moreover, it was reported in GeneReviews that CMT1A (70%-80% of all CMT1) involves duplication of *PMP22* gene. So we speculated that the duplications of CDS1-4 in the *PMP22* gene were the causative mutations for patient P57 which was confirmed by real-time PCR.

Two samples (P88, P89) were tissues of spontaneous abortion. After normalization analysis, we identified the genders of the two samples were all female and detected a duplication of chromosome 10 in patient P88, the gender ratio was about 1.42. We also detected a duplication of chromosome 9 in patient P89, the gender ratio was about 1.31 (S4 Table). Based on karyotype analysis, patient P88 was diagnosed with trisomy 10 syndrome and patient P89 was diagnosed with trisomy 9 syndrome. So our results were consistent with the results of karyotype analysis. Clinical features of patient P90 were lordosis of lower lip, crooked teeth, mental retardation and she was talkative but the words fail to convey the idea. Previously, two microdeletions (del 6p21.1; del 17p11.2) were detected using WGS. As is well known, Smith-Magenis Syndrome (SMS) is caused by mutations in the *RAI1* gene on chromosome 17p11.2 (SMS, OMIM# 1882290). Due to analyzed regions of the array did not include chromosome 6, we couldn’t detect the mutation of del 6p21.1,but we detected a microdeletion in chromosome 17 (16773072–20222149) and the gender ration was about 0.55 (S4 Table), which was consistent with the result of WGS.

### Variants Confirmation

**Fig 5:**
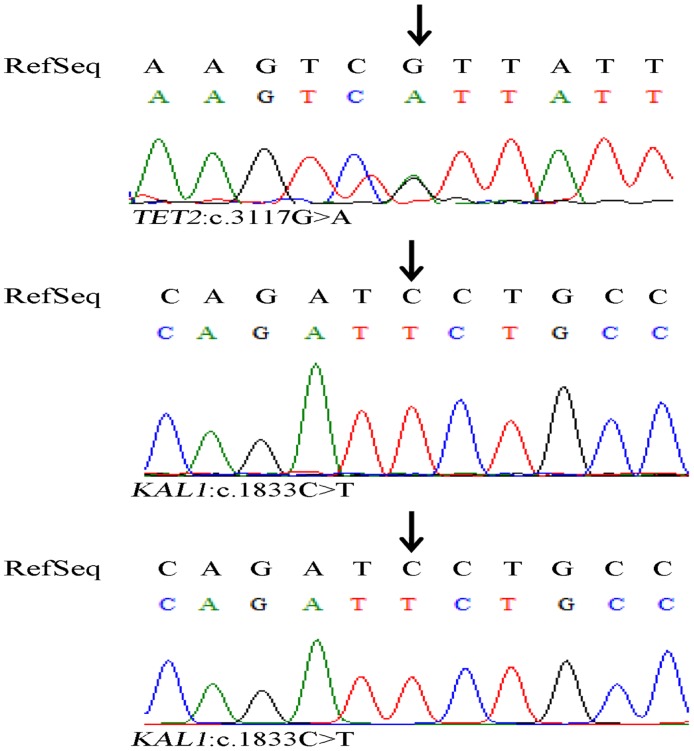
Confirmation of inconsistent genotypes detected by targeted NGS and Snp Array using Sanger sequencing. (A) A heterozygous substitution of G with A was confirmed in the *TET2* gene in sample S1-1. (B) A Homozygous substitution of C with T was confirmed in the *KAL1* gene in sample S1-1. (C) A Homozygous substitution of C with T was confirmed in the *KAL1* gene in sample S4.

We did validation for the inconsistent genotypes detected by targeted NGS and SNP Array respectively. The inconsistent genotype of T in locus of 14851554 in Y chromosome was detected in both S2 and S3 by SNP Array, but it was not detected by targeted NGS (Table 2). It is obviously wrong to detect a genotype in the Y-chromosome of a normal female. In addition, we designed primers (S5 Table) and try to target the site, but we got no PCR products. Moreover, it turned out to be that all the genotypes of other three inconsistent loci detected by targeted NGS were consistent with that of Sanger sequencing (Fig 5), which suggested that our method provides high accuracy.

For 5 patients (P2, P49, P53, P58 and P76), in order to verify the results of our method and determine whether the disease-causing mutations were inherited from father or mother of the proband, 6 primer pairs were designed (S5 Table) to target the mutated loci and the 6 mutations of pedigrees were confirmed by Sanger sequencing. A hemizygous *COL4A5* gene missense mutation (c.1769A->C, p.K590T, MIM# 301050) was detected in patient P2 with Alport Syndrome. Sanger sequencing confirmed this mutation in the proband and showed that his mother was a carrier of the heterozygous mutation, which was accorded with X-linked inheritance pattern (Figure A in S1 Fig). A heterozygous *AARS* gene missense mutation (c.2042G>T, p.G681V, MIM# 613287) was detected in patient P49 with autosomal dominant CMT disease type 2N. Sanger sequencing confirmed this mutation in the proband and showed that his father did not carry the mutation and although his mother was a carrier of the heterozygous mutation but she was asymptomatic (Figure B in S1 Fig). There were two possible assumptions, the mutation was pathogenic but the inheritance was incomplete in his mother or the mutation was non-pathogenic and the disease of the proband was caused by other unknown reasons. We detected two different mutations in patient P53 with CMT disease. One was a heterozygous gene mutation (c.1039-2A>G, MIM# 609260) in the AG splicing region of *MFN2* gene. This mutation could cause autosomal dominant CMT disease type 2A2. Sanger sequencing showed that his mother was not a carrier of this mutation and although his father was a carrier of the heterozygous mutation, he was asymptomatic, so the mutation was not the cause of the disease of patient P53 (Figure C in S1 Fig). The other was a hemizygous *GJB1* gene missense mutation (c.265C>G, p.L89V, MIM# 302800). It was reported that mutation c.266T>C in *GJB1* gene could cause X-linked CMT disease type 1, indicating that the amino acid of this site was conservative [22]. Sanger sequencing showed that his father was not a carrier and his mother was a carrier of the heterozygous mutation, which was accorded with X-linked inheritance pattern, so this was a suspected pathogenic mutation (Figure D in S1 Fig). A homozygous *FGA* gene frame shift mutation (c.1368delC, p.T457Rfs*27, MIM# 202400) was detected in patient P58 with autosomal recessive Congenital Afibrinogenemia. Sanger sequencing showed that his parents were all carriers of the heterozygous mutation (Figure E in S1 Fig), so the mutation was the disease-causing mutation. A heterozygous *OTX2* gene nonsense mutation (c.538C>T, p.Q180*, MIM# 610125) was detected in patient P76 with autosomal dominant syndromic Microphthalmia type 5. However, his parents did not carry the mutation according to Sanger sequencing (Figure F in S1 Fig). Maybe the mutation of proband was caused by mosaicism of germ cells or spontaneous mutation.

Using quantitative real-time PCR, heterozygous duplications of CDS1-4 in the *PMP22* gene of patient P57 were confirmed. A total of 4 primer pairs (S5 Table) were designed to target CDS1-4 of *PMP22* gene. The relative quantification (RQ) of CDS1–4 in patient P57 were 1.4965, 1.5674, 1.5513 and 1.5306 respectively, ~150% of the RQ in control sample (C1) (Fig 4B), suggesting that there were heterozygous duplications of CDS1–4 in the *PMP22* gene of this patient. The results showed complete consistency of targeted NGS and real-time PCR.

## Discussion

Targeted NGS has equal quality to Sanger sequencing in detecting mutations in disease-specific genes, so it can replace Sanger sequencing in diagnostic test [23]. Moreover, the feasibility of using targeted NGS sequencing for genetic diagnosis of inherited diseases has been demonstrated [24]. In this study, we used targeted NGS to mainly capture the CDS of 2,181 genes associated with 561 Mendelian diseases and detected variants for clinical diagnosis. The 561 Mendelian diseases belonged to 17 classifications of diseases, which included genetic bone disease, genodermatosis, hereditary neuropathy, neurocutaneous syndrome, hereditary neuromuscular disease, hereditary muscular disease, hereditary metabolic disease, immune system disorder, endocrine system disease, digestive system disease, respiratory disease, cardiovascular and cerebrovascular disease, hereditary kidney disease, genetic blood disease, hereditary ophthalmopathy, cancer and carcinoid syndrome and multisystemic syndrome. The test of the evaluation showed that the method could detect SNVs with high accuracy and good stability even in the case of low average depth which was only about 80-fold. Moreover, using the pipeline of bioinformatic analysis, we could not only detect SNVs but also detect Indels, CNVs, microdeletions and microduplications of chromosomes. Six candidate mutations in 5 pedigrees and duplications in *PMP22* gene were confirmed by Sanger sequencing or real-time PCR, indicating the accuracy and reliability of our method in clinical diagnosis of Mendelian diseases.

For 90 clinical cases, we at first found candidate disease-causing genes in our list of 561 disorders based on the clinical diagnosis and used targeted NGS to find pathogenic mutations in the candidate genes. If the mutation was uncertain based on the results of information analysis and annotated databases we would draw the distribution diagram of reads aligning to reference for the mutated site with a single base resolution. Sanger sequencing or real-time PCR would be performed if the mutation still could not be confirmed. However, the pathogenic mutations of some cases (P47, P48, P59, P65, P77 and P86) were not in the candidate genes. We would find candidate mutations in the other genes in the target regions and backward infer the disease. Clinical features of patient P47 were low sodium and low potassium and the clinical diagnosis was suspected Bartter Syndrome (BS). Two heterozygous *CFTR* gene mutations (c.1116+1G>A; c.3062C>T, p.P1021L, MIM# 219700) were detected in patient P47. c.1116+1G>A was a splicing mutation which could cause abnormal splice of mRNA and change primary structure of protein. It was included in the database of cystic fibrosis (http://www.genet.sickkids.on.ca/app). c.3062C>T was a missense mutation and Pro1021 was very conservative. Moreover, it was reported that the exchange of proline residue could influence the function of chloride channel [25]. So the compound heterozygous mutations were the causing mutations of cystic fibrosis for this patient. Clinical features of patient P48 were potassium and sodium deficiency, electrolyte instability and mild iodine poisoning. The clinical diagnosis was also suspected BS. A homozygous *CFTR* gene missense mutation (c.2909G>A, p.G970D, MIM# 219700) was detected in this patient. The incidence of this mutation was very low and it was harmful annotated by SIFT (http://sift.jcvi.org/) and PolyPhen-2 (http://genetics.bwh.harvard.edu/pph2/). It was reported that the mutation could cause Cystic Fibrosis [26] and it was recessive heredity. So c.2909G>A was the pathogenic mutation of patient P48. Clinical features of patient P59 were hemolytic anemia and poor body immunity after birth. She was suspected to suffer from congenital Dyserythropoietic Anemia. Two heterozygous *PKLR* gene mutations (c.661G>A, p.D221N; c.1528C>T, p.R510*, MIM# 266200) were detected in patient P59. c.661G>A was a missense mutation and the other was a nonsense mutation. They were both harmful annotated by SIFT and PolyPhen-2. It was reported that the two mutations could cause Pyruvate Kinase Deficiency [27–28]. So the two mutations were causative mutations for the case. We did not get the clinical feature of P65 and the clinical diagnosis was suspected Glycogen Storage Disease. A homozygous *GALT* gene missense mutation (c.1043A>G, p.D348G, MIM# 230400) was detected in this patient. The incidence of this mutation was very low and it was harmful annotated by SIFT and PolyPhen-2. Based on annotated databases, mutations in the *GALT* gene could cause Galactosemia with an autosomal recessive mode of inheritance, so c.1043A>G was the suspected pathogenic mutation for this case. Clinical features of patient P77 were slow retarded physical development, mental retardation, flattened nose, hepatosplenomegaly, sparse teeth, large head, thick valgus lips, thick eyebrows, claw hand and feet, stubby fingers, kyphosis and short stature, which were very similar to the phenotypes of mucopolysaccharidosis. Two heterozygous *GNPTAB* gene mutations (c.3565C>T, p.R1189*; c.2590_2591insG, p.E864Gfs*4, MIM# 252500, MIM# 252600) were detected in patient P77. It was reported that c.3565C>T could lead to Mucolipidosis II Alpha/Beta and Mucolipidosis III Alpha/Beta [29]. Although there was no literature reported about the mutation of c.2590_2591insG, the incidence was very low according to annotated databases. So the compound heterozygous mutations were pathogenic mutations of this patient. Clinical features of patient P86 were recurrent multiple pathogen infection (respiratory, digestive, blood), eczema history, idiopathic thrombocytopenic purpura, enlargement of cervical lymph node and submandibular lymphatic nodes. The disease was onset after 3 months old. She was suspected to get Wiskott-Aldrich Syndrome or X-linked Severe Combined Immunodeficiency. Two heterozygous *RAG1* gene mutations (c.874T>C, p.S292P; c.1328G>A, p.R443K, MIM# 603554) were detected in patient P86. The two mutations were both harmful annotated by database of SIFT and PolyPhen-2 and the incidence was very low. Mutations in the *RAG1* gene could cause autosomal recessive Omenn Syndrome based on annotated databases, therefore, the compound heterozygous mutations were suspected causative mutations of this patient. So our method could not only find pathogenic mutations and confirm the clinical diagnose but also find misdiagnosis of clinical cases. There were 15 samples with negative results. We thought that the reason might be clinical misdiagnosis or the disease-causing gene was not included in the list of 2,181 genes. Cases that remain unsolved by our method can be considered for more time-intensive WES or WGS studies.

Having a newborn with a genetic disease is a disaster for a family. Carrier screening can estimate the risk of offspring with a genetic disease, which plays an important role in fertility guidance and may induce the birth rate of newborns with genetic diseases. Our method can be used as a promising tool for screening asymptomatic couples especially who once have newborns with one of the 561 Mendelian diseases such as the three couples of suspected carriers.

Due to the target regions mainly include CDS of 2,181 genes, there are some limitations of our method. First, deep intronic mutations and complex rearrangements may not be detected. For example, we detected a heterozygous *PRF1* gene synonymous mutation (c.1620A>G, p.Q540Q, MIM# 603553) in patient P63 who was diagnosed with Familial Hemophagocytic Lymphohistiocytosis (FHL). It has been reported that the mutation could cause FHL2 with an autosomal recessive inheritance [30]. However, maybe due to the limitation, there were no other disease-causing mutations were detected in the *PRF1* gene of this patient. The mutation of c.1620A>G was a suspected disease-causing mutations, which may constitute compound heterozygous mutations combining with other unknown mutations and cause the disease. Second, we may only detect chromosomal microdeletions and microduplications in the list of detection related to the target regions or part of a microdeletion or microduplication in the list. For example, we only detect one mutation and a part (16773072–20222149) of the mutated region of del 17p11.2 in patient P90, partly reflecting SMS.

In summary, we have presented a tool for diagnostic testing that combines capture array and NGS of a panel of 2,181 genes known to be associated with 561 Mendelian diseases. Results of the evaluation showed that our method had high accuracy and stability in detecting disease-causing mutations. The high throughput and speed of our method have been proved in a previous study [9]. So the technology can be used for diagnostic testing, providing effective basis for the clinical diagnosis or genetic counseling of 561 Mendelian diseases.

## Supporting Information

S1 Fig Confirmation of mutations in 5 pedigrees by Sanger sequencing. A hemizygous *COL4A5* gene missense mutation (c.1769A>C) in patient P2 was confirmed (Figure A). A heterozygous *AARS* gene missense mutation (c.2042G>T) in patient P49 was confirmed (Figure B). A heterozygous gene mutation (c.1039-2A>G) in the AG splicing region of *MFN2* gene in patient P53 was confirmed (Figure C). A hemizygous *GJB1* gene missense mutation (c.265C>G) in patient P53 was confirmed (Figure D). A homozygous *FGA* gene frame shift mutation (c.1368delC) in patient P58 was confirmed (Figure E). A heterozygous *OTX2* gene nonsense mutation (c.538C>T) in patient P76 was confirmed (Figure F).
(TIF)

S1 Table Overview of sequencing data of our method. (DOC)

S2 Table The abbreviations of the names of diseases. (DOC)

S3 Table The list of 561 Mendelian diseases. (DOC)

S4 Table Results of normalization analysis of three patients (P88, P89 and P90). We detected a microduplication of chromosome 10 in patient P88, the gender ratio was about 1.42. We detected a microduplication of chromosome 9 in patient P89, the gender ratio was about 1.31. We detected a microdeletion in chromosome 17 (16773072–20222149) in patient P90, the gender ration was about 0.55.
(DOC)

S5 Table Primer pairs designed for validation of mutations by Sanger sequencing or real-time PCR. (DOC)
