# Correction: Targeted Next-Generation Sequencing for Clinical Diagnosis of 561 Mendelian Diseases

**DOI:** 10.1371/journal.pone.0139258

**Published:** 2015-09-22

**Authors:** Yanqiu Liu, Xiaoming Wei, Xiangdong Kong, Xueqin Guo, Yan Sun, Jianfen Man, Lique Du, Hui Zhu, Zelan Qu, Ping Tian, Bing Mao, Yun Yang

There is an additional affiliation for the fifth author. Yan Sun is also affiliated with Department of Biology, University of Copenhagen, Copenhagen, Denmark.
