# Correction: Targeted Next-Generation Sequencing for Clinical Diagnosis of 561 Mendelian Diseases

**DOI:** 10.1371/journal.pone.0148154

**Published:** 2016-01-28

**Authors:** Yanqiu Liu, Xiaoming Wei, Xiangdong Kong, Xueqin Guo, Yan Sun, Jianfen Man, Lique Du, Hui Zhu, Zelan Qu, Ping Tian, Bing Mao, Yun Yang

S3, S4 and S5 Tables appear incorrectly in the published article. Please see the correct tables below.

## Supporting Information

S3 Table The list of 561 Mendelian diseases. (XLSX)

S4 Table Results of normalization analysis of three patients (P88, P89 and P90). We detected a microduplication of chromosome 10 in patient P88, the gender ratio was about 1.42. We detected a microduplication of chromosome 9 in patient P89, the gender ratio was about 1.31. We detected a microdeletion in chromosome 17 (16773072–20222149) in patient P90, the gender ration was about 0.55.
(DOC)

S5 Table Primer pairs designed for validation of mutations by Sanger sequencing or real-time PCR. (DOC)
