# Supplementary figures and images for: Targeted Next-Generation Sequencing for Clinical Diagnosis of 561 Mendelian Diseases
Source: PLoS One. 2015 Aug 14;10(8):e0133636. doi: 10.1371/journal.pone.0133636 (PMC4537117; doi:10.1371/journal.pone.0133636)

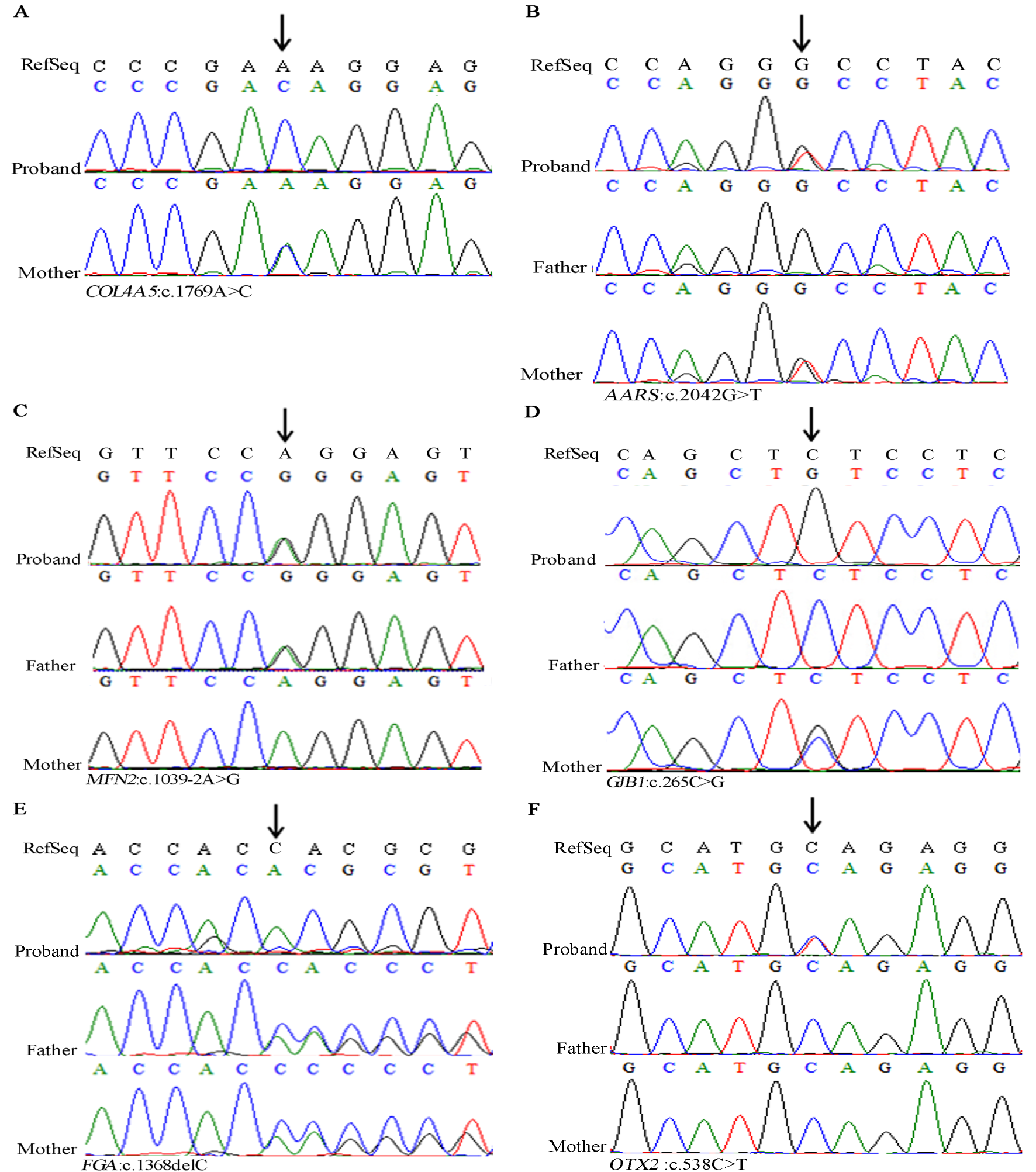

Supplement: S1 Fig — A hemizygous COL4A5 gene missense mutation (c.1769A>C) in patient P2 was confirmed (Figure A). A heterozygous AARS gene missense mutation (c.2042G>T) in patient P49 was confirmed (Figure B). A heterozygous gene mutation (c.1039-2A>G) in the AG splicing region of MFN2 gene in patient P53 was confirmed (Figure C). A hemizygous GJB1 gene missense mutation (c.265C>G) in patient P53 was confirmed (Figure D). A homozygous FGA gene frame shift mutation (c.1368delC) in patient P58 was confirmed (Figure E). A heterozygous OTX2 gene nonsense mutation (c.538C>T) in patient P76 was confirmed (Figure F). (TIF) [file pone.0133636.s001.tif]
