# Supplementary material for: Targeted Next-Generation Sequencing for Clinical Diagnosis of 561 Mendelian Diseases
Source: PLoS One. 2015 Aug 14;10(8):e0133636. doi: 10.1371/journal.pone.0133636 (PMC4537117; doi:10.1371/journal.pone.0133636)
Supplement: S1 Table — (DOC) [file pone.0133636.s002.doc]

S1 Table. Overview of sequencing data of our method.

| **Sample** | **Reads mapped to hg19** | **Reads mapped to target region** | **Mean depth(x)** | **Fraction of target region covered with at least 20X (%)** | **Coverage of target region(%)** |
| --- | --- | --- | --- | --- | --- |
| S1-1 | 9,378,147 | 7,094,604 | 79.49 | 96.98 | 99.7 |
| S1-2 | 10,067,239 | 7,556,266 | 84.28 | 97.03 | 99.68 |
| S1-3 | 9,311,989 | 7,017,753 | 78.29 | 96.7 | 99.66 |
| S1-4 | 11,823,374 | 8,883,750 | 98.99 | 97.63 | 99.69 |
| S1-5 | 20,996,343 | 16,064,508 | 180.71 | 98.71 | 99.7 |
| S2 | 23,611,300 | 17,368,695 | 191.6 | 98.5 | 99.59 |
| S3 | 24,828,309 | 18,266,816 | 201.8 | 98.49 | 99.58 |
| S4 | 24,782,268 | 18,323,594 | 202.19 | 98.7 | 99.78 |
| P1 | 21,936,868 | 16,231,572 | 177.66 | 98.3 | 99.48 |
| P2 | 20,883,001 | 15,493,904 | 172.96 | 98.83 | 99.81 |
| P3 | 27,862,424 | 20,410,109 | 225.22 | 98.57 | 99.49 |
| P4 | 22,858,774 | 17,166,012 | 191.09 | 98.43 | 99.52 |
| P5 | 24,287,455 | 18,196,277 | 202.09 | 98.69 | 99.72 |
| P6 | 18,387,929 | 13,722,760 | 152.23 | 98.37 | 99.66 |
| P7 | 24,406,905 | 18,232,074 | 202.83 | 98.45 | 99.51 |
| P8 | 24,310,124 | 18,059,755 | 199.99 | 98.42 | 99.51 |
| P9 | 22,296,004 | 16,621,242 | 184.77 | 98.55 | 99.66 |
| P10 | 18,967,319 | 14,163,489 | 157.51 | 98.33 | 99.64 |
| P11 | 18,401,906 | 13862026 | 155.29 | 98.45 | 99.65 |
| P12 | 20,441,910 | 15,479,537 | 173.18 | 98.46 | 99.63 |
| P13 | 18,315,716 | 13,518,313 | 149.05 | 98.09 | 99.46 |
| P14 | 20,513,532 | 15,322,041 | 170.67 | 98.35 | 99.47 |
| P15 | 20,315,828 | 15,135,589 | 168.25 | 98.35 | 99.51 |
| P16 | 19,709,521 | 14,574,996 | 161.42 | 98.49 | 99.68 |
| P17 | 19,090,259 | 14,164,733 | 157.03 | 98.48 | 99.68 |
| P18 | 19,689,052 | 14,658,606 | 162.6 | 98.43 | 99.67 |
| P19 | 19,989,075 | 15,039,532 | 168.21 | 98.39 | 99.51 |
| P20 | 19,910,187 | 14,772,992 | 164.05 | 98.51 | 99.67 |
| P21 | 21,350,911 | 16,037,363 | 178.84 | 98.62 | 99.7 |
| P22 | 21,965,709 | 16,318,126 | 180.88 | 98.64 | 99.69 |
| P23 | 20,892,355 | 15,667,644 | 174.83 | 98.66 | 99.69 |
| P24 | 22,019,227 | 16,316,856 | 180.56 | 98.42 | 99.48 |
| P25 | 21,426,584 | 15,806,791 | 174.49 | 98.54 | 99.67 |
| P26 | 19,342,646 | 14,379,616 | 159.66 | 98.45 | 99.65 |
| P27 | 20,141,531 | 14,970,189 | 165.98 | 98.46 | 99.64 |
| P28 | 20,869,745 | 15,529,470 | 172.37 | 98.58 | 99.68 |
| P29 | 19,269,497 | 14,339,298 | 158.82 | 98.46 | 99.68 |
| P30 | 22,247,010 | 16,308,114 | 180.33 | 98.27 | 99.46 |
| P31 | 19,595,210 | 14,430,121 | 159.9 | 98.45 | 99.68 |
| P32 | 34,579,529 | 25,140,275 | 277.43 | 98.7 | 99.52 |
| P33 | 24,610,165 | 18,444,547 | 204.8 | 98.76 | 99.7 |
| P34 | 19,556,657 | 14,943,903 | 168.56 | 97.68 | 99.5 |
| P35 | 17,408,102 | 13,350,187 | 151.21 | 90.34 | 99.63 |
| P36 | 21,914,739 | 16,584,512 | 186 | 96.74 | 99.53 |
| P37 | 17,286,585 | 13,339,068 | 149.03 | 98.65 | 99.71 |
| P38 | 19,607,719 | 15,167,441 | 169.92 | 98.8 | 99.75 |
| P39 | 18961,004 | 14,571,937 | 162.2 | 98.41 | 99.47 |
| P40 | 24,313,078 | 18,050,651 | 199.01 | 98.82 | 99.71 |
| P41 | 19,807,522 | 14,879,359 | 165.36 | 98.38 | 99.51 |
| P42 | 20,782,634 | 15,495,057 | 171.77 | 98.64 | 99.71 |
| P43 | 20,959,398 | 10,536,248 | 175.45 | 98.72 | 99.8 |
| P44 | 23,862,996 | 17,927,380 | 199.98 | 98.92 | 99.82 |
| P45 | 24,235,495 | 18,201,286 | 203.7 | 98.66 | 99.58 |
| P46 | 23,097,179 | 17,453,798 | 195.44 | 98.53 | 99.51 |
| P47 | 24,083,779 | 18,383,338 | 204.32 | 98.74 | 99.51 |
| P48 | 24,687,218 | 18,275,529 | 201.61 | 98.73 | 99.68 |
| P49 | 19,765,436 | 14,607,715 | 162.71 | 98.49 | 99.58 |
| P50 | 20,324,701 | 15,079,300 | 168.05 | 98.59 | 99.59 |
| P51 | 19,884,084 | 14,712,611 | 163.9 | 98.57 | 99.6 |
| P52 | 25,529,841 | 19,126,951 | 213.82 | 98.67 | 99.57 |
| P53 | 16,650,149 | 12,475,275 | 138.93 | 98.27 | 99.65 |
| P54 | 19,628,121 | 14,866,695 | 166.75 | 98.49 | 99.66 |
| P55 | 15,256,070 | 11,817,729 | 132.01 | 98.21 | 99.48 |
| P56 | 25,278,135 | 19,116,358 | 212.15 | 98.65 | 99.67 |
| P57 | 23,314,727 | 17,523,807 | 195.84 | 96.27 | 99.73 |
| P58 | 21,338,617 | 15,940,410 | 176.72 | 98.31 | 99.48 |
| P59 | 16,861,751 | 12,814,589 | 145.02 | 93.05 | 99.48 |
| P60 | 24,896,741 | 19,267,874 | 215.64 | 98.76 | 99.54 |
| P61 | 14,905,333 | 11,187,164 | 124.77 | 97.17 | 99.65 |
| P62 | 17,682,080 | 13,592,530 | 151.09 | 98.69 | 99.73 |
| P63 | 22,812,965 | 17,036,688 | 189.14 | 98.33 | 99.47 |
| P64 | 22,525,219 | 16,842,437 | 186.91 | 98.46 | 99.46 |
| P65 | 22,096,644 | 16,585,107 | 185.44 | 96.47 | 99.73 |
| P66 | 24,716,076 | 18,172,805 | 200.95 | 98.67 | 99.69 |
| P67 | 18,151,398 | 13,534,294 | 150.25 | 98.4 | 99.66 |
| P68 | 17,260,842 | 13,620,534 | 159.59 | 98.44 | 99.71 |
| P69 | 22,331,788 | 16,891,913 | 189.37 | 98.36 | 99.73 |
| P70 | 25,777,704 | 19,262,896 | 214.8 | 98.75 | 99.6 |
| P71 | 28,242,406 | 20,461,697 | 223.79 | 98.65 | 99.65 |
| P72 | 9,244,369 | 6,925,841 | 77.43 | 95.08 | 99.57 |
| P73 | 20,302,660 | 15,615,310 | 176.14 | 98.31 | 99.43 |
| P74 | 28,344,706 | 20,046,718 | 217.01 | 98.78 | 99.67 |
| P75 | 14,052,513 | 10,043,636 | 108.8 | 97.85 | 99.46 |
| P76 | 18,187,632 | 13,728,693 | 153.95 | 98.39 | 99.67 |
| P77 | 25,880,466 | 19,245,144 | 214.07 | 98.65 | 99.53 |
| P78 | 17,982,975 | 13,361,920 | 147.72 | 98.32 | 99.63 |
| P79 | 20,351,929 | 15,224,407 | 169.22 | 98.26 | 99.46 |
| P80 | 19,427,782 | 14,615099 | 164.21 | 98.47 | 99.6 |
| P81 | 21,940,674 | 16,478,986 | 183.47 | 98.43 | 99.5 |
| P82 | 15,009,845 | 11,318,677 | 126.71 | 98.14 | 99.65 |
| P83 | 19,093,915 | 14,636,669 | 162.57 | 98.68 | 99.71 |
| P84 | 19,092,217 | 14,042,232 | 155.87 | 98.4 | 99.59 |
| P85 | 19,351,774 | 14,717,907 | 165.31 | 97.85 | 99.53 |
| P86 | 17,028,105 | 13,144,344 | 148.57 | 97.5 | 99.4 |
| P87 | 19,353,104 | 14,798,296 | 166.79 | 94.61 | 99.67 |
| P88 | 17,390,401 | 13,440,347 | 153.21 | 96.52 | 99.46 |
| P89 | 16,001,214 | 12,441,320 | 142.81 | 97.03 | 99.49 |
| P90 | 19,219,711 | 14,685,694 | 165.42 | 97.74 | 99.49 |
