# Supplementary material for: Targeted Next-Generation Sequencing for Clinical Diagnosis of 561 Mendelian Diseases
Source: PLoS One. 2015 Aug 14;10(8):e0133636. doi: 10.1371/journal.pone.0133636 (PMC4537117; doi:10.1371/journal.pone.0133636)
Supplement: S2 Table — (DOC) [file pone.0133636.s003.doc]

S2 Table. The [abbreviation](javascript:void(0);)s of the names of diseases.

| **Abbreviation** | **Disease** |
| --- | --- |
| AxD | Alexander Disease |
| AS | Alport Syndrome |
| APS-1 | Autoimmune Polyendocrine Syndrome,type 1 |
| ADPKD | Autosomal Dominant Polycystic Kidney Disease |
| ARCI | Autosomal Recessive Congenital Ichthyosis |
| BS | Bartter Syndrome |
| CMT | Charcot-Marie-Tooth Disease |
| CDA | Congenital Dyserythropoietic Anemia |
| CdLS | Cornelia de Lange Syndrome |
| CF | Cystic Fibrosis |
| GS | Gitelman Syndrome |
| FHL | Familial Hemophagocytic Lymphohistiocytosis |
| HSAN | Hereditary Sensory and Autonomic Neuropathy |
| IP | Incontinentia pigmenti |
| IBD deficiency | Isobutyryl-CoA dehydrogenase deficiency |
| EE | Ethylmalonic Encephalopathy |
| SCAD deficiency | Short-Chain Acyl-CoA Dehydrogenase Deficiency |
| LPG | Lipoprotein Glomerulopathy |
| MMA | Methylmalonic Acidemia |
| OCA | Oculocutaneous Albinism |
| OS | Omenn Syndrome |
| MCPH | Primary Autosomal Recessive Microcephaly |
| PCD | Primary Ciliary Dyskinesia |
| PK Deficiency | Pyruvate Kinase Deficiency |
| SMS | Smith-Magenis Syndrome |
| SLSN | Senior-Loken Syndrome |
| SRNS | Steroid-Resistant Nephrotic Syndrome |
| WAS | Wiskott-Aldrich Syndrome |
| X-SCID | X-Linked Severe Combined Immunodeficiency |
| XLA | X-Linked Agammaglobulinemia |
