# Supplementary material for: Correction: Targeted Next-Generation Sequencing for Clinical Diagnosis of 561 Mendelian Diseases
Source: PLoS One. 2016 Jan 28;11(1):e0148154. doi: 10.1371/journal.pone.0148154 (PMC4731064; doi:10.1371/journal.pone.0148154)
Supplement: S4 Table — We detected a microduplication of chromosome 10 in patient P88, the gender ratio was about 1.42. We detected a microduplication of chromosome 9 in patient P89, the gender ratio was about 1.31. We detected a microdeletion in chromosome 17 (16773072–20222149) in patient P90, the gender ration was about 0.55. (DOC) [file pone.0148154.s002.doc]

S4 Table. Results of normalization analysis of P88, P89 and P90.

| **Disease** | **Region** | **P88** | | **P89** | | **P90** | |
| --- | --- | --- | --- | --- | --- | --- | --- |
|  |  | **Gender ratio**^a^ | **status** | **Gender ratio**^a^ | **status** | **Gender ratio**^a^ | **status** |
| 12q14 microdeletion syndrome | chr12:65071919-68645525 | 0.873462 | normal | 0.882799 | normal | 1.058086 | normal |
| 15q13.3 microdeletion syndrome | chr15:30910306-32445407 | 1.028557 | normal | 0.913206 | normal | 0.918945 | normal |
| 15q24 recurrent microdeletion syndrome | chr15:74412643-75972911 | 0.980518 | normal | 0.905083 | normal | 1.029036 | normal |
| 15q26 overgrowth syndrome | chr15:99357970-102521392 | 1.036957 | normal | 0.941379 | normal | 1.038994 | normal |
| 16p11.2 microduplication syndrome | chr16:29606852-30199855 | 0.921766 | normal | 0.925029 | normal | 1.085589 | normal |
| 16p11.2-p12.2 microdeletion syndrome | chr16:21512062-30199854 | 0.962386 | normal | 0.946356 | normal | 1.068027 | normal |
| 16p11.2-p12.2 microduplication syndrome | chr16:21475060-29284077 | 0.971687 | normal | 0.95124 | normal | 1.064102 | normal |
| 16p13.11 recurrent microdeletion or microduplication | chr16:14986684-16486684 | 0.992038 | normal | 0.93529 | normal | 1.03914 | normal |
| 17q21.31 recurrent microdeletion syndrome | chr17:43705166-44294406 | 1.025659 | normal | 0.920844 | normal | 0.997661 | normal |
| 1p36 microdeletion syndrome | chr1:10001-12840259 | 0.986448 | normal | 0.947672 | normal | 1.06001 | normal |
| 1q21.1 recurrent microdeletion or microduplication | chr1:146533376-147883376 | 1.052363 | normal | 0.923976 | normal | 0.988372 | normal |
| 1q21.1 susceptibility locus for Thrombocytopenia Absent Radius syndrome | chr1:145386506-145748067 | 0.97219 | normal | 0.879239 | normal | 0.961418 | normal |
| 22q11 deletion or duplication syndrome | chr22:19009792-21452445 | 0.985779 | normal | 0.912153 | normal | 1.063214 | normal |
| 22q13 deletion syndrome | chr22:51045516-51187844 | 1.000491 | normal | 1.015458 | normal | 1.046327 | normal |
| 2p15-16.1 microdeletion syndrome | chr2:59285696-61819815 | 0.940692 | normal | 0.916262 | normal | 1.048837 | normal |
| 2p21 microdeletion syndrome | chr2:44410451-44589584 | 0.963535 | normal | 0.904901 | normal | 1.064922 | normal |
| 2q33.1 deletion syndrome | chr2:196925121-205206939 | 0.96812 | normal | 0.931498 | normal | 1.015576 | normal |
| 2q37 monosomy | chr2:239969863-240322643 | 0.994032 | normal | 0.944212 | normal | 1.044629 | normal |
| 3q29 microdeletion or microduplication syndrome | chr3:195726835-197344663 | 0.901356 | normal | 0.947655 | normal | 1.106299 | normal |
| 7q11.23 duplication syndrome | chr7:72744455-74142672 | 0.932632 | normal | 0.88111 | normal | 1.066567 | normal |
| 8p23.1 deletion or duplication syndrome | chr8:8100055-11764629 | 1.032791 | normal | 0.95497 | normal | 1.096511 | normal |
| 9q subtelomeric deletion syndrome | chr9:140513443-140730578 | 0.934914 | normal | 1.358219 | normal | 1.02986 | normal |
| Angelman syndrome Type1 | chr15:22749354-28438266 | 0.991373 | normal | 0.934848 | normal | 1.038078 | normal |
| Angelman syndrome Type2 | chr15:23619912-28438266 | 0.991009 | normal | 0.933405 | normal | 1.034809 | normal |
| ATR-16 syndrome | chr16:60001-834372 | 0.929842 | normal | 0.944187 | normal | 1.127276 | normal |
| AZFa | chrY:14352761-15154862 | 0 | normal | 0 | normal | 0 | normal |
| Charcot-Marie-Tooth syndrome type1A | chr17:14097915-15470903 | 1.077074 | normal | 0.971311 | normal | 0.963532 | normal |
| Cri du Chat Syndrome | chr5:10001-12533304 | 1.012131 | normal | 0.934632 | normal | 1.053526 | normal |
| Early-onset Alzheimer disease with cerebral amyloid angiopathy | chr21:27252860-27543446 | 0.929241 | normal | 0.937008 | normal | 1.004172 | normal |
| Familial Adenomatous Polyposis | chr5:112043201-112181936 | 1.093362 | normal | 0.96202 | normal | 1.024451 | normal |
| Miller-Dieker Syndrome | chr17:1-2588909 | 0.929114 | normal | 0.9028 | normal | 1.043855 | normal |
| NF1-microdeletion Syndrome | chr17:29107097-30263321 | 0.946281 | normal | 0.941346 | normal | 1.025648 | normal |
| Pelizaeus-Merzbacher disease | chrX:103031438-103047547 | 0.982679 | normal | 0.917984 | normal | 1.054998 | normal |
| Smith-Magenis Syndrome | chr17:16773072-20222149 | 1.035588 | normal | 0.903284 | normal | 0.54783 | deletion |
| Potocki-Shaffer syndrome | chr11:43994800-46052450 | 1.009246 | normal | 0.9412 | normal | 1.064137 | normal |
| Renal cysts and diabetes | chr17:34815072-36215917 | 1.019237 | normal | 0.918565 | normal | 1.01506 | normal |
| Rubinstein-Taybi Syndrome | chr16:3775055-3930121 | 1.013941 | normal | 0.948575 | normal | 0.944131 | normal |
| Sotos syndrome | chr5:175724636-177052116 | 0.949981 | normal | 0.956242 | normal | 1.039909 | normal |
| Steroid sulphatase deficiency | chrX:6455812-8133195 | 0.975052 | normal | 0.941034 | normal | 1.04786 | normal |
| 11p13 deletion syndrome | chr11:31806339-32457087 | 1.040063 | normal | 0.995843 | normal | 1.032506 | normal |
| Wolf-Hirschhorn syndrome | chr4:1569197-2110236 | 0.962811 | normal | 1.000397 | normal | 1.13383 | normal |
| Xp11.22-linked intellectual disability | chrX:53401070-53683275 | 1.017831 | normal | 0.92016 | normal | 0.887755 | normal |
| Xp11.22-p11.23 microduplication | chrX:48334549-52117661 | 0.983511 | normal | 0.933577 | normal | 0.972684 | normal |
| Xq28 duplication | chrX:153287263-153363188 | 1.490229 | normal | 0.902291 | normal | 1.025081 | normal |
| Xq28 microduplication | chrX:153624563-153881853 | 0.937995 | normal | 1.009479 | normal | 1.020298 | normal |
| Chr1 | chr1:0-249250621 | 0.975999 | normal | 0.923601 | normal | 1.028743 | normal |
| Chr 2 | chr2:0-243199373 | 0.97933 | normal | 0.912917 | normal | 1.028716 | normal |
| Chr 3 | chr3:0-198022430 | 0.935031 | normal | 0.907403 | normal | 1.00932 | normal |
| Chr 4 | chr4:0-191154276 | 0.967262 | normal | 0.94246 | normal | 1.032839 | normal |
| Chr 5 | chr5:0-180915260 | 0.983628 | normal | 0.935875 | normal | 1.025102 | normal |
| Chr 6 | chr6:0-171115067 | 0.921948 | normal | 0.887805 | normal | 0.985457 | normal |
| Chr 7 | chr7:0-159138663 | 0.969785 | normal | 0.916634 | normal | 1.026849 | normal |
| Chr 8 | chr8:0-146364022 | 0.988624 | normal | 0.936213 | normal | 1.018105 | normal |
| Chr 9 | chr9:0-141213431 | 0.91613 | normal | 1.310585 | duplication | 1.029443 | normal |
| Chr 10 | chr10:0-135534747 | 1.421047 | duplication | 0.854507 | normal | 1.00786 | normal |
| Chr 11 | chr11:0-135006516 | 0.996189 | normal | 0.954487 | normal | 1.028709 | normal |
| Chr 12 | chr12:0-133851895 | 0.993979 | normal | 0.924915 | normal | 1.003229 | normal |
| Chr 13 | chr13:0-115169878 | 0.969576 | normal | 0.900144 | normal | 1.02072 | normal |
| Chr 14 | chr14:0-107349540 | 1.015249 | normal | 0.97752 | normal | 0.988312 | normal |
| Chr 15 | chr15:0-102531392 | 1.010911 | normal | 0.940793 | normal | 1.019595 | normal |
| Chr 16 | chr16:0-90354753 | 0.976597 | normal | 0.940923 | normal | 1.026196 | normal |
| Chr 17 | chr17:0-81195210 | 0.982672 | normal | 0.909672 | normal | 1.00646 | normal |
| Chr 18 | chr18:0-78077248 | 1.012233 | normal | 0.924001 | normal | 1.014145 | normal |
| Chr 19 | chr19:0-59128983 | 0.968837 | normal | 0.939016 | normal | 1.002876 | normal |
| Chr 20 | chr20:0-63025520 | 0.990922 | normal | 0.918569 | normal | 1.045005 | normal |
| Chr 21 | chr21:0-48129895 | 1.02641 | normal | 0.95071 | normal | 1.053619 | normal |
| Chr 22 | chr22:0-51304566 | 0.985762 | normal | 0.953585 | normal | 1.04312 | normal |
| Chr X | chrX:0-155270560 | 0.960588 | normal | 0.939177 | normal | 0.997955 | normal |
| Chr Y | chrY:0-59373566 | 0 | normal | 0 | normal | 0 | normal |
