# Supplementary material for: Correction: Targeted Next-Generation Sequencing for Clinical Diagnosis of 561 Mendelian Diseases
Source: PLoS One. 2016 Jan 28;11(1):e0148154. doi: 10.1371/journal.pone.0148154 (PMC4731064; doi:10.1371/journal.pone.0148154)
Supplement: S5 Table — (DOC) [file pone.0148154.s003.doc]

S5 Table. Primer pairs designed in validation of mutations by Sanger sequencing or real-time PCR.

| **Primer name** | **Sequence (5’-3’ direction)** | **Length (bp)** | **Annealing (°C)** |
| --- | --- | --- | --- |
| USP9Y-1413_F | AGTTGCCAGGTATTAAATGACAGTTC | 0 | 60 |
| USP9Y-1413_R | GAGATTCTTCCATGAAAATGTTAGTTAA |
| TET2-3117_F | GCTGGGGTGTGGCTATCAAG | 0 | 60 |
| TET2-3117_R | CCTCCCCAGAAGGACACTCA |
| KAL1-1833_F | ACACCTTCTCCAGTCGCCTAA | 678 | 60 |
| KAL1-1833_R | GAAGTTGCGGGGAAGACG |
| COL4A5-1769_F | TGTTTGATTCCTTGACTCTTCCTG | 477 | 60 |
| COL4A5-1769_R | GTTTATCTCAGCATCAGTCCCATC |
| AARS-2042_F | CATACTGATGCCCTTGACTTTGCAC | 393 | 60 |
| AARS-2042_R | TGACCTGTCTACTCTGCCCCTC |
| MFN2-1039-2_F | AGGCTTTCAAGTGAGGATGTTTG | 490 | 60 |
| MFN2-1039-2_R | ACCAGGCTGTCTATGTGGTTCT |
| GJB1-265_F | CTATGACCAATTCTTCCCCATCTCC | 291 | 60 |
| GJB1-265_R | GCATAGCCAGGGTAGAGCAGATA |
| FGA-1368_F | AGAGGTGTCAGGAAATGTAAGTCC | 363 | 60 |
| FGA-1368_R | GGTGAGAAGAAACCTGGGAATG |
| OTX2-538_F | CAGCAGAATGGAGGTCAAAACA | 434 | 60 |
| OTX2-538_R | CCATATCCCTGGGTGGAAAGAG |
| PMP22-CDS1_F | AACTCCGCTGAGCAGAACTTG | 107 | 60 |
| PMP22-CDS1_R | GCTGACGATCGTGGAGACG |
| PMP22-CDS2_F | CAATGGATCGTGGGCAATG | 100 | 60 |
| PMP22-CDS2_R | CGTTTGGTGATGATGAGAAACAG |
| PMP22-CDS3_F | CGATCATCTTCAGCATTCTGTCTC | 93 | 60 |
| PMP22-CDS3_R | GGAAGATTCCAGTGATGTAAAACCT |
| PMP22-CDS4_F | CTGTGCGTGATGAGTGCTGC | 135 | 60 |
| PMP22-CDS4_R | GATGACACCGCTGAGAAGGG |
